# Ceramides: a potential therapeutic target in pulmonary emphysema

**DOI:** 10.1186/1465-9921-14-96

**Published:** 2013-10-01

**Authors:** Jeroen Tibboel, Irwin Reiss, Johan C de Jongste, Martin Post

**Affiliations:** 1 Physiology and Experimental Medicine Program, The Hospital for Sick Children, 555 University Avenue, Toronto, Ontario M5G 1X8, Canada; 2 Department of Laboratory Medicine and Pathobiology, University Of Toronto, Toronto, Canada; 3 Department of Pediatrics, Erasmus University Medical Center – Sophia Children’s Hospital, Rotterdam, the Netherlands

**Keywords:** Elastase, Sphingolipids, Ceramide, Lung function

## Abstract

**Background:**

The aim of this manuscript was to characterize airway ceramide profiles in a rodent model of elastase-induced emphysema and to examine the effect of pharmacological intervention directed towards ceramide metabolism.

**Methods:**

Adult mice were anesthetized and treated with an intratracheal instillation of elastase. Lung function was measured, broncho-alveolar lavage fluid collected and histological and morphometrical analysis of lung tissue performed within 3 weeks after elastase injection, with and without sphingomyelinase inhibitors or serine palmitoyltransferase inhibitor. Ceramides in broncho-alveolar lavage (BAL) fluid were quantified by tandem mass spectrometry.

**Results:**

BAL fluid showed a transient increase in total protein and IgM, and activated macrophages and neutrophils. Ceramides were transiently upregulated at day 2 after elastase treatment. Histology showed persistent patchy alveolar destruction at day 2 after elastase installation. Acid and neutral sphingomyelinase inhibitors had no effect on BAL ceramide levels, lung function or histology. Addition of a serine palmitoyltransferase inhibitor ameliorated lung function changes and reduced ceramides in BAL.

**Conclusions:**

Ceramides were increased during the acute inflammatory phase of elastase-induced lung injury. Since addition of a serine palmitoyltransferase inhibitor diminished the rise in ceramides and ameliorated lung function, ceramides likely contributed to the early phase of alveolar destruction and are a potential therapeutic target in the elastase model of lung emphysema.

## Background

Chronic Obstructive Pulmonary Disease (COPD) is a common and increasing source of morbidity and mortality in the developed world [1] and is associated with a large cost burden [2]. Pulmonary emphysema, an important component of COPD, is caused by permanent destruction of alveoli, airflow obstruction and lung hyperinflation, leading to a decreased lung function and breathlessness. The pathogenesis is related to smoke exposure, but why only a minority of all smokers develops emphysema remains unclear. Reports suggest that inflammation is partly responsible. The inflammatory infiltrate seen in emphysema is similar to that found in infection and correlates to the extent of emphysema [3], while the degree of cell death correlates to the amount of inflammation [4]. In humans, airway inflammation persists for many years after smoking cessation [5]. Sphingolipids are important structural components of biological membranes and have recently been shown to serve as messenger molecules in cell proliferation, apoptosis, cell contact and adhesion, endothelial barrier function and during inflammation [6-14]. Ceramide is the central molecule in the sphingolipid pathway [15] and is formed either *de novo* from the condensation of palmitate with serine via the activity of a serine palmitoyltransferase (SPT), by the salvage pathway via sphingosine or by degradation of sphingomyelin by sphingomyelinase (SMase) [16]. Ceramide is degraded by ceramidase to sphingosine which can be phosphorylated to sphingosine-1-phosphate (S1P) [17]. Ceramide and S1P form a rheostat [17,18] whereby ceramide stimulates apoptosis and cell cycle arrest while S1P stimulates cell survival and proliferation. Sphingolipid metabolism has been shown to be altered in a variety of diseases, including cystic fibrosis [19,20] and asthma [21]. Ceramide has been shown to trigger apoptosis in an experimental mouse model of emphysema [22], and increased levels of apoptosis have been found in the lungs of patients with severe cigarette-induced emphysema [23]. Increased ceramide levels have also been shown to influence surfactant production [24] and activity [25]. Since ceramide levels are increased in the lungs of patients with smoke-induced emphysema [22] ceramide upregulation might be an important pathogenetic element in emphysema development. We investigated ceramide profiles in the lungs and examined the effect of pharmacological interventions targeting SMases and SPT in an animal model of elastase-induced emphysema.

## Methods

### Animals

*A*nimals were obtained from Charles River (St. Constant, Quebec, Canada) and animal studies were conducted according to criteria established by the Canadian Council for Animal Care and approved by the Animal Care and Use Committee of the Hospital for Sick Children, Toronto, ON, Canada. Female adult C57BL/6N mice, weighing between 22 and 25 grams, were used for all experiments.

### Elastase-induced lung injury

Porcine pancreatic elastase (Type I, aqueous suspension, ≥4.0 units/mg protein, Calbiochem, EMD biosciences, USA) was dissolved in sterile saline to create a volume for tracheal instillation of 100 μl per mouse with a concentration of 4.8 Units/100 g bodyweight. Animals were anesthetized with 3% isoflurane and intraperitoneal (*ip*) administration of 75 mg/kg ketamine (75 mg/kg) and 5 mg/kg xylazine (5 mg/kg). Following induction of anesthesia, a 25 G intubation tube was inserted past the vocal cords and 100 μl of elastase instilled into the trachea. Control animals were treated similarly, but received sterile saline instead of elastase. BAL was collected from mice at t = 1, 2, 3, 4, 5, 8, 14 and 21 days after elastase instillation to measure sphingolipids and inflammatory markers.

### Lung function measurements

At day 21 following the instillation of elastase, the Flexivent rodent ventilator (Scireq, Montreal, Canada) was used to assess lung function as previously published [26].

### Broncho-alveolar lavage

Lungs were infused through the endotracheal tube with 3x 600 μl sterile saline, followed by withdrawal [27,28]. The collected fluid was centrifuged at 1400*g* for 8 min. The supernatant and remaining lung tissue was collected in siliconized eppendorf tubes and stored at −80°C for mass spectrometry analysis.

### Histology of the lungs

Following lung function measurements, histology and morphometry of the lungs was performed as previously described [26].

### Measurement of ceramides

Ceramide levels in BAL and remaining lung tissue were measured by tandem mass spectrometry as previously described [26]. The analysis was performed by the Analytical facility for Bioactive Molecules, The Hospital for Sick Children, Toronto, Canada.

### Ceramide inhibitor experiments

Desipramine (acid SMase inhibitor, 20 mg/kg bodyweight), zoledronic acid (acid SMase inhibitor, 0.1 mg/kg bodyweight), sphingolactone (neutral SMase inhibitor, 1mg/kg bodyweight) (Sigma–Aldrich, St. Louis, MO), and myriocin (SPT inhibitor, 1mg/kg bodyweight) (Cayman Chemicals, Ann Arbor, MI) were administered via *ip* injection 2 hours before elastase instillation and 6, 24, 48 and 72 hours after elastase instillation. Each sphingolipid inhibitor experiment consisted of 4 groups: 1) control mice, 2) control mice treated with an *ip* injection of vehicle, 3) elastase-treated mice with an *ip* injection of vehicle only and 4) elastase-treated mice with an *ip* injection of sphingolipid inhibitor dissolved in the appropriate vehicle. Half of the number of the mice in each group was sacrificed at day 2 after elastase instillation to measure sphingolipid levels and inflammatory markers in BAL. The other mice underwent lung function measurements at day 14 after elastase instillation before histology and morphometry.

### Immunofluorescent (IF) staining

IF was performed according to a previously published protocol with slight modification [29]. Tissue sections were de-waxed in xylene, rehydrated using decreasing ethanol series (100% to 70%), before being washed in 1xPBS/0.03% (vol/vol) Triton-100-X. Tissue permeabilization was achieved by boiling in 10 mM sodium citrate (pH 6) for 15 minutes at 95°C, and cooled for 30 minutes in room temperature. Nonspecific antibody binding was blocked by incubation with a solution containing 10% (vol/ vol) normal donkey serum (Jackson ImmunoResearch, Cedarlane Laboratories, Burlington, Ontario) and 1% (vol/vol) bovine serum albumin in PBS at room temperature for 1 hour. The sections were washed and incubated for 1 hour with 1:100 diluted anti-ceramide monoclonal IgM antibodies (Glycobiotech, Borstel, Germany). The slides were washed again and stained for 30 min with a 1:200 diluted Cy3-labeled donkey anti-mouse IgM (Jackson ImmunoResearch,). After rinsing, the samples were mounted with 4,6-diamidino-2-phenylindole (DAPI) mounting medium (Vector, Burlington (ON)) and analyzed on a Leica fluorescence microscope.

### Western blotting

Lung tissues were lysed, protein content measured and aliquots (50 ug protein) were separated on 4-12% Bis-Tris precast polyacrylamide gels (Invitrogen, Cat. NP0322BOX) and transferred to PVDF membranes. After blocking with 5% (w/v) skim milk in TBST (20 mM Tris, 137 mM NaCl, 0.1% Tween 20) membranes were incubated with either goat anti-acid ceramidase antibody (1:500 dilution; T-20 Santa Cruz Biotechnology, CA), rabbit anti-acid sphingomyelinase (1:2000 dilution; H-181 Santa Cruz Biotehcnology, CA) or goat anti-neutral ceramidase (1:500 dilution; S-20 Santa Cruz Biotechnology) overnight in 4°C. The next day the membranes were washed TBST and incubated with either horseradish peroxidase–conjugated donkey anti-goat (1:20.000 dilution) or goat anti-rabbit (1:20.000 dilution) in 5% (w/v) skim milk in TBST at RT for 1–2 hrs. After several washes with TBST, protein bands were visualized using an enhanced chemiluminescence detection kit. Band densities were quantified using Scion Image software (Version 1.6, National Institutes of Health, Bethesda, MD, USA). Equal protein loading was confirmed by immunoblotting for β-actin of same membrane.

### Statistics

All values are presented as mean ± standard error of the mean assuming normal distribution (Sigmaplot 11 for Windows). Differences were assessed by Student’s t test or, for comparison of more than two groups, by two-way analysis of variance followed by Holm-Sidak comparison test. Significance was inferred where P<0.05.

## Results

### BAL analysis

**Figure 1:**
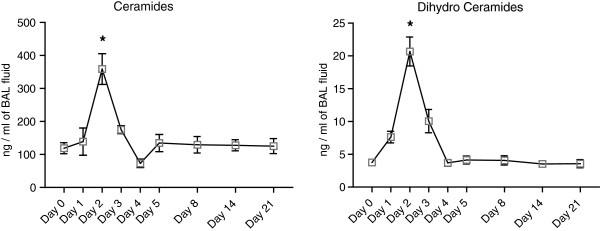
**Ceramide and dihydroceramide levels in BAL fluid during the first 21 days after elastase injection.** Results represent a total of n=4 mice per group for days 1, 3 and 4, n=8 mice per group for days 8, 14, 21 and n=12 mice per group for days 0, 2 and 5. * = p<0.05.

**Figure 2:**
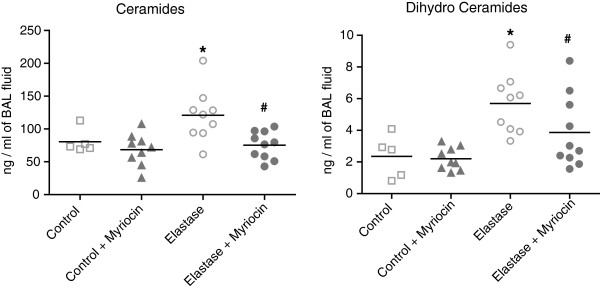
**Ceramide and dihydroceramide levels in BAL fluid at day 2 after elastase injection and myriocin treatment.** Results represent a total of n=5 mice for control, n=9 mice for control + myriocin, n=9 mice for elastase and n=10 mice for elastase + myriocin group. * = p<0.05 when compared to Control. ^#^ p=<0.05 when compared to elastase.

**Figure 3:**
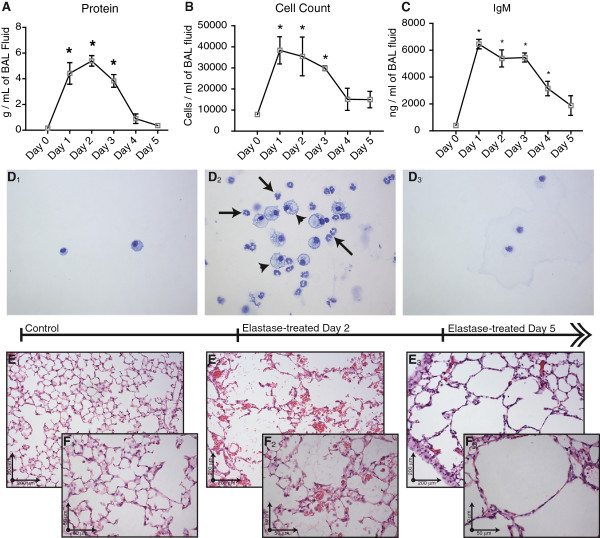
**Biochemical measurements in BAL fluid.** Protein levels **(A)** and cell count **(B)** at day 1, 2 and 3 after elastase injection. IgM levels **(C)** measured by ELISA. Cytospin slides **(D)** and haemotoxilin-eosin stained histological slides from control mice (E1 at 200x magnification, F1 at 400x magnification) and elastase-treated mice at 2 days after elastase injection (E2 at 200x magnification, F2 at 400x magnification) and 5 days after elastase injection (E3 at 200x magnification, F3 at 400x magnification). Arrows indicate neutrophils, arrowheads indicate macrophages. Graphs represent an n=4 for each group. * = p<0.05.

**Figure 4:**
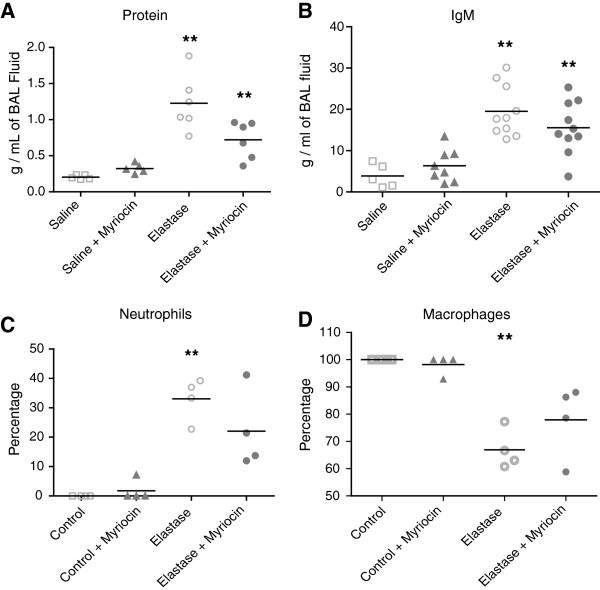
Protein levels (A), IgM content (B), neutrophil (C) and macrophage (D) percentages in BAL fluid at day 2 after elastase instillation and myriocin treatment.

A 4-fold increase in total ceramide and dihydroceramide levels was found at day 2 after elastase instillation when compared to saline-treated controls (Figure 1 and Table 1). Ceramide levels were 20-fold greater than dihydroceramide levels. Specifically, long chain and very long chain ceramides (Cer16:0, Cer22:0, Cer24:0 and Cer24:1) were increased. All ceramides and dihydroceramides returned to baseline levels within 5 days after elastase treatment. No difference in ceramide levels were noted in remaining lung tissue samples (data not shown), therefore only BAL was analysed in subsequent experiments. Neither desipramine, zoledronic acid or sphingolactone treatment altered ceramide levels (Table 2), but myriocin treatment ameliorated elastase-induced increases in multiple ceramides (Cer22:0, Cer24:0, Cer24:1), and dihydroceramides (18:0 and 24:0) (Figure 2, Table 3). Protein and IgM content (Figure 3A, C) and cell counts (Figure 3B) in BAL were increased at day 1, 2 and 3 after elastase injection. Cytospins from the same BAL fluid showed an increase in normal and activated “foamy” macrophages, and neutrophils, which normalized by day 5 after elastase injection (Figure 3D). Neither desipramine, zoledronic acid nor sphingolactone treatment had any effect on the above mentioned parameters. Myriocin treatment decreased BAL protein levels, shifted the neutrophil/macrophage balance towards neutrophils (Figure 4), but had no significant effect on IgM.

**Table 1:** Ceramides after elastase injection

**Ceramide**	**Day 0**	**Day 1**	**Day 2**	**Day 3**	**Day 4**	**Day 5**	**Day 8**	**Day 14**	**Day 21**
Ceramide 16:0	45.63 ± 33.52	32.04 ± 14.16	110.54 ± 57.84 *	48.52 ± 3.87	21.91 ± 6.73	52.84 ± 1.86	40.97 ± 21.74	46.66 ± 27.52	38.04 ± 20.54
Ceramide 18:0	2.47 ± 0.68	3.55 ± 1.71	5.50 ± 1.55*	2.97 ± 0.48	1.38 ± 0.23	1.86 ± 0.61	2.20 ± 1.18	2.11 ± 0.72	2.46 ± 1.58
Ceramide 20:0	1.39 ± 0.40	1.92 ± 0.77	4.25 ± 1.98*	1.86 ± 0.36	0.79 ± 0.16	1.14 ± 0.40	1.41 ± 0.79	1.30 ± 0.41	1.32 ± 0.83
Ceramide 22:0	9.60 ± 2.47	14.12 ± 7.82	29.75 ± 11.28*	16.31 ± 2.80	6.66 ± 2.30	9.00 ± 2.85	10.89 ± 5.74	10.13 ± 2.12	10.40 ± 5.40
Ceramide 24:0	34.83 ± 12.23	34.97 ± 20.00	112.76 ± 47.82*	44.75 ± 6.09	19.09 ± 6.43	36.70 ± 16.82	40.97 ± 20.87	37.58 ± 9.04	41.64 ± 19.19
Ceramide 24:1	24.89 ± 8.47	52.02 ± 37.77	95.90 ± 41.08*	60.25 ± 9.65*	23.04 ± 9.75	33.03 ± 12.09	32.90 ± 20.18	30.01 ± 8.14	31.41 ± 16.8
Dihydro Ceramide 18:0	0.53 ± 0.24	2.01 ± 0.40*	1.49 ± 0.53	1.51 ± 0.44	0.75 ± 0.34	0.36 ± 0.10	0.48 ± 0.31	0.40 ± 0.16	0.33 ± 0.15
Dihydro Ceramide 24:0	2.17 ± 0.68	4.64 ± 1.14	10.74 ± 4.32*	3.77 ± 1.95	1.72 ± 0.34	1.83 ± 0.85	2.21 ± 0.99	1.85 ± 0.49	1.83 ± 0.85

BAL fluid from 12 mice per group was collected and processed using LC-MS/MS. Data are expressed as Mean + SEM ng/ml BAL fluid. *p<0.05, when compared to control animals at the same time-point.

**Table 2:** Ceramides after Desipramine, Zoledronic Acid or Sphingolactone treatment

**Sphingolipid**	**Control (n=5)**	**Control + Sph (n=9)**	**Control + ZA (n=9)**	**Control + Des (n=10)**	**Elastase (n=5)**	**Elastase + Sph (n=9)**	**Elastase + ZA (n=9)**	**Elastase + Des (n=10)**
Ceramide 16:0	23.39 ± 2.92	25.55 ± 1.23	26.07 ± 2.85	32.70 ± 3.12 *	48.38 ± 4.46 *	53.07 ± 12.34 *^$^	44.47 ± 11.93 *^$^	63.15 ± 13.15 *^#$^
Ceramide 18:0	2.61 ± 0.54	2.74 ± 0.30	2.83 ± 0.20	4.01 ± 0.70	4.88 ± 0.60 *	5.66 ± 1.62 *^$^	4.43 ± 0.97 *^$^	7.45 ± 1.32 *^#$^
Ceramide 20:0	1.30 ± 0.34	1.24 ± 0.12	1.32 ± 0.14	1.97 ± 0.27	2.56 ± 0.37 *	2.86 ± 0.78 *^$^	2.44 ± 0.37 *^$^	4.07 ± 0.82 *^#$^
Ceramide 22:0	7.95 ± 2.41	7.37 ± 0.70	9.75 ± 1.29	14.17 ± 1.89 *	20.01 ± 4.39 *	20.43 ± 5.74 *^$^	19.80 ± 4.73 *^$^	33.90 ± 7.71 *^#$^
Ceramide 24:0	33.63 ± 13.22	25.92 ± 2.66	42.31 ± 6.19	56.01 ± 6.39 *	86.39 ± 22.72 *	71.40 ± 16.60 *^$^	84.40 ± 16.74 *^$^	145.10 ± 41.79 *^#$^
Ceramide 24:1	19.09 ± 6.27	17.97 ± 0.90	22.81 ± 4.10	33.25 ± 4.01 *	68.45 ± 19.42 *	64.66 ± 16.75 *^$^	70.20 ± 16.85 *^$^	113.70 ± 26.87 *^#$^
Dihydro Ceramide 18:0	0.32 ± 0.07	0.38 ± 0.23	0.39 ± 0.30	0.42 ± 0.08	0.88 ± 0.21 *	1.02 ± 0.35 *^$^	0.86 ± 0.20 *^$^	1.76 ± 0.24 *^#$^
Dihydro Ceramide 24:0	1.57 ± 0.67	1.67 ± 0.75	2.02 ± 1.11	2.32 ± 1.78	6.54 ± 1.78 *	6.28 ± 2.49 *^$^	6.29 ± 1.52 *^$^	13.11 ± 5.03 *^#$^

BAL fluid was collected and processed using LCMS. Data are expressed as Mean + SEM in ng/ml BAL fluid. Des = Desimaprimine, ZA = Zoledronic Acid, Sph = Sphingolactone. *p<0.05 when compared to Control. ^#^ p<0.05 when compared to Elastase. ^$^p<0.05 when compared to control group with the same inhibitor (Elastase + Sph vs. Control + Sph, Elastase + ZA vs. Control + ZA, Elastase + Des vs. Control + Des).

**Table 3:** Ceramides after Myriocin treatment

**Sphingolipid**	**Control (n=5)**	**Control + Myriocin (n=9)**	**Elastase (n=9)**	**Elastase + Myriocin (n=10)**
Ceramide 16:0	30.01 ± 6.65	22.23 ± 7.95	41.04 ± 18.77	21.69 ± 4.91^[]^
Ceramide 18:0	3.13 ± 0.58	2.86 ± 0.75	4.00 ± 1.89	2.61 ± 0.60^[]^
Ceramide 20:0	1.94 ± 0.47	1.93 ± 0.43	2.73 ± 1.18	2.02 ± 0.43
Ceramide 22:0	7.97 ± 1.67	7.99 ± 3.42	15.13 ± 7.96*	9.11 ± 3.02^[]^
Ceramide 24:0	37.34 ± 9.00	33.24 ± 13.50	77.75 ± 44.65*	39.72 ± 13.00^[]^
Ceramide 24:1	24.66 ± 7.60	17.33 ± 8.02	41.48 ± 20.83*	19.44 ± 6.27^[]^
Dihydro Ceramide 18:0	0.38 ± 0.21	0.37 ± 0.20	0.70 ± 0.27*	0.44 ± 0.17^[]^
Dihydro Ceramide 24:0	1.97 ± 1.18	1.83 ± 0.65	6.12 ± 3.94*	3.42 ± 2.21^[]^

BAL fluid was collected and processed using LCMS. Data are expressed as Mean + SEM in ng/ml BAL fluid. *p<0.05 when compared to Control. ^[]^ p<0.05 when compared to Elastase + Myriocin.

### Enzyme expression

**Figure 5:**
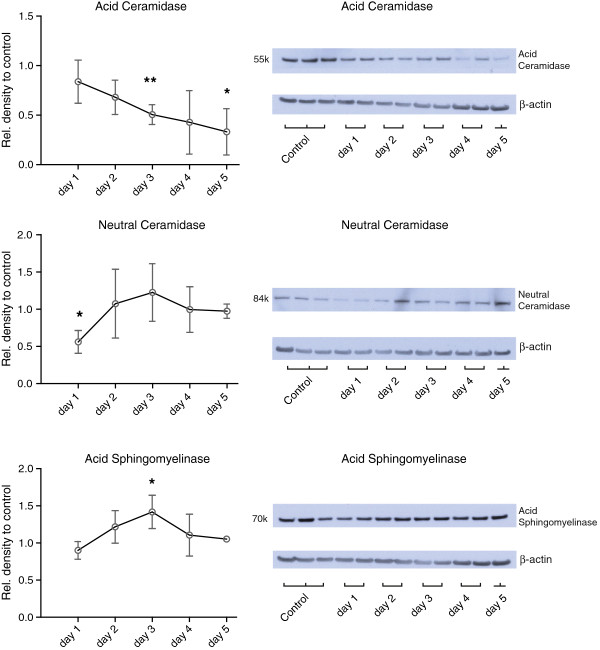
**Densitometric expression analysis of acid sphingomyelinase, acid- and neutral ceramidase proteins accompanied by representative western blots**. All data is expressed as mean ± SEM. n=4 for day 1, 2 and 3, n=3 for day 4 and 5. * = p<0.05 and ** = p<0.001 compared to control levels (which were set at 1).

Western blot analysis of enzymes partaking in ceramide metabolism showed decreased acid ceramidase levels during the first 5 days after the elastase treatment, reaching significance at day 3 and 5. Neutral ceramidase levels were only significantly downregulated at day 1 after elastase injection. Acid sphingomyelinase levels were only significantly upregulated at day 3 after elastase instillation (Figure 5).

### Lung function

**Figure 6:**
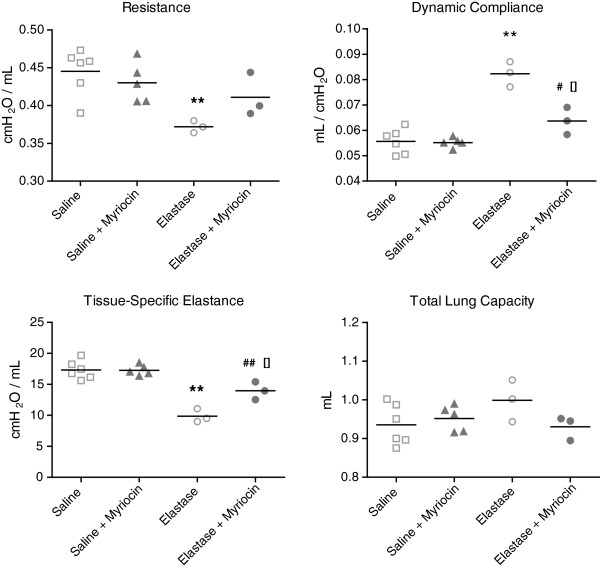
**Lung function 2 weeks after elastase injection in myriocin-treated mice.** Results represent n=6 mice for the saline and saline + myriocin treated groups, n=3 mice for the elastase-treated and elastase-treated + myriocin group, and an n=25 mice for the control group. Data are expressed in mean ± SEM. ** = p<0.05 when compared to the saline and saline + myriocin group. ^[]^ = p<0.05 when compared to the elastase-treated group.

Flexivent lung function measurements of elastase-treated mice showed significant reductions in resistance and tissue-specific elastance and an increase in dynamic compliance compared to controls (Figure 6, Table 4). Neither desipramine, zoledronic acid nor sphingolactone had any effect on lung function in elastase-treated animals compared to vehicle-treated elastase-exposed mice (Table 4). Elastase-treated mice receiving myriocin showed a significant decrease in compliance and increase in tissue elastasticity compared to control elastase-exposed mice (even with a limited number of mice). Myriocin also prevented the decrease in resistance, normally seen in elastase-treated mice, compared to vehicle-treated elastase-exposed mice (Figure 6, Table 5).

**Table 4:** Invasive lung function measurements after desimaprimine, zoledronic acid and sphingolactone treatment

**Parameter**	**Unit**	**Control (n=9)**	**Elastase (n=10)**	**Elastase + desimaprimine (n=5)**	**Elastase + zoledronic acid (n=4)**	**Elastase + sphingolactone (n=4)**
Resistance (R)	cmH_2_O/mL	0.67 ± 0.07	0.52 ± 0.03*	0.47 ± 0.02 *	0.49 ± 0.01*	0.54 ± 0.05*
Compliance (C)	mL/cmH_2_O	0.032 ± 0.004	0.052 ± 0.004*	0.066 ± 0.007*	0.067 ± 0.011*	0.054 ± 0.007*
Airway Resistance (Rn)	cmH_2_O/mL	0.32 ± 0.03	0.23 ± 0.03*	0.22 ± 0.02*	0.25 ± 0.01*	0.21 ± 0.02*
Tissue Elasticity (H)	cmH_2_O/mL	32.4 ± 4.2	16.9 ± 2.3*	13.2 ± 1.3*	13.9 ± 2.8*	15.5 ± 2.7*
Total lung capacity (A)	mL	0.70 ± 0.05	0.78 ± 0.04	0.88 ± 0.02	0.89 ± 0.07	0.77 ± 0.03
Inspiratory Capacity (B) from zero pressure	mL	1.14 ± 0.09	1.30 ± 0.11*	1.47 ± 0.028*	1.57 ± 0.19*	1.29 ± 0.09*
Static Compliance (Cst)	mL/cmH_2_O	0.078 ± 0.008	0.094 ± 0.009*	0.108 ± 0.002*	0.115 ± 0.014*	0.094 ± 0.008*

During invasive lung function measurements, an average for each individual mouse was calculated from 4 accepted (coefficient of determination: COD>0.95) measurements for each parameter. * p<0.05 when compared to Control.

**Table 5:** Invasive lung function measurements after Myriocin treatment

**Parameter**	**Unit**	**Control (n=6)**	**Control + Myriocin (n=5)**	**Elastase (n=3)**	**Elastase + Myriocin (n=3)**
Resistance	cmH_2_O/mL	0.44 ± 0.03	0.43 ± 0.03	0.37 ± 0.01*	0.41 ± 0.03
Compliance	mL/cmH_2_O	0.056 ± 0.005	0.055 ± 0.002	0.082 ± 0.005*	0.063 ± 0.005^#^^[]^
Airway Resistance	cmH_2_O/mL	0.22 ± 0.01	0.22 ± 0.02	0.16 ± 0.03*	0.18 ± 0.01^#^
Tissue Elasticity	cmH_2_O/mL	17.3 ± 1.5	17.3 ± 0.8	9.9 ± 1.1*	14.0 ± 1.4^# []^
Total lung capacity	mL	0.93 ± 0.05	0.95 ± 0.03	0.99 ± 0.05	0.93 ± 0.03
Inspiratory Capacity from zero pressure	mL	1.47 ± 0.08	1.49 ± 0.07	1.42 ± 0.06	1.36 ± 0.11
Static Compliance	mL/cmH_2_O	0.105 ± 0.006	0.106 ± 0.005	0.104 ± 0.004	0.097 ± 0.009

During invasive lung function measurements, an average for each individual mouse was calculated from 4 accepted (coefficient of determination: COD>0.95) measurements for each parameter. * p<0.05 when compared to Control. ^#^ p<0.05 when compared to Control + Myriocin. ^[]^ p<0.05 when compared to Elastase.

### Histology and morphometry

**Figure 7:**
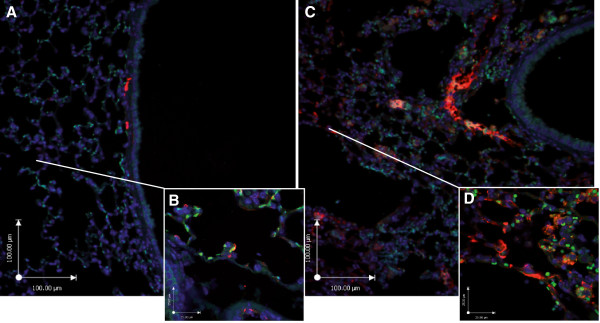
**Immunofluorescent images of ceramide expression.** Control (**A** at 100x magnification, **B** at 400x magnification), elastase-treated mice 2 days after elastase injection (**C** at 100x magnification, **D** at 400x magnification). Ceramide (red), cell nuclei (DAPI blue) and autofluorescence (green).

Histological analysis of the lung at day 2 following elastase injection revealed the presence of erythrocytes in the alveolar spaces and massive influx of neutrophils and macrophages (Figure 3E). Alveolar enlargement was present from day 2 onwards. Erythrocytes and most of the inflammatory cells were cleared by day 5 after elastase injection. Immunofluorescent staining for ceramide showed increased positive reactivity in the epithelial lining and inflammatory cells in elastase-treated mice 2 days after elastase injection compared to controls (Figure 7). Three weeks after elastase installation we observed a patchy pattern of alveolar destruction leading to enlarged airspaces. This was reflected in a significant increase in mean linear intercept and decrease in alveolar number. Mice treated with SMase inhibitors or STP inhibitor showed no significant signs of histological recovery compared to elastase-treated controls (Table 6).

**Table 6:** Morphometry after elastase instillation and inhibitor treatment

**Parameter**	**Unit**	**Control (n=10)**	**Elastase (n=10)**	**Elastase + desimaprimine (n=4)**	**Elastase + zoledronic acid (n=4)**	**Elastase + sphingolactone (n=4)**	**Elastase + Myriocin (n=6)**
Mean Linear Intercept	μm	42.2 (40.8-43.2)	141.8 (133.3-152.0)	119.8 (105.8-147.4)	137.0 (108.1-170.8)	129.5 (114.8-158.3)	126.5 (85.6-164.4)
Tissue-vs-Air ratio	count	19.9 ± 2.0	15.3 ± 2.8*	13.7 ± 1.3*	15.7 ± 2.4*	15.9 ± 2.1*	18.5 ± 3.1

During invasive lung function measurements, an average for each individual mouse was calculated from 4 accepted (coefficient of determination: COD>0.95) measurements for each parameter. * p<0.05 when compared to Control.

## Discussion

In this study we showed that intratracheal elastase caused a transient inflammatory response with influx of erythrocytes and inflammatory cells in the alveolar spaces, combined with an increase in multiple ceramide and dihydroceramide species in BAL fluid which peaked at day 1 and 2 and returned to control levels 3 days after elastase instillation. Ceramide expression was primarily localized to the epithelium and to inflammatory cells. Sphingomyelinase inhibitors did not prevent or reduce the effects of intratracheal elastase. In contrast, SPT inhibition reduced sphingolipid and protein levels and ameliorated lung function changes after elastase instillation.

Apoptosis of alveolar cells has been shown to play a crucial role in the development of emphysema [30-32]. Petrache *et al*. showed that direct intratracheal instillation of a synthetic short chain ceramide caused emphysema-like defects [33] and observed increased ceramide levels in the lungs of individuals with cigarette smoke-induced emphysema [33]. Our study shows for the first time that ceramide levels are increased in the first few days following the intratracheal instillation of elastase in mice. Earlier studies have described an increase in apoptosis in the elastase-induced emphysema model [34-37]. Long chain ceramides (Cer16:0, Cer18:0) have been shown in cancer cells to exhibit anti-proliferative and pro-apoptotic effects [38]. Our study shows that long chain ceramides are increased a few days after elastase instillation and we speculate that they were responsible for triggering apoptosis. Long chain ceramides have also been shown to increase the permeability of the lung endothelium after cigarette-smoke [39] and thereby enhance endothelial leakage leading to edema formation [40]. In our model we found evidence of significant edema formation at day 2 and 3 after elastase, as measured by increased IgM and protein levels in the BAL fluid. Our observation of long chain ceramides being increased at those time-points is suggestive of a causal role of ceramides in our model. The elastase-induced emphysema model does not mimic the low-grade, long-term inflammatory pathophysiology of smoke-induced pulmonary emphysema in patients [41]. However, it is an excellent model to accurately reproduce the histological damage seen in emphysematous lungs and to evaluate the effect of interventions. The short developmental time of the model when compared to genetic or cigarette smoke models makes it easier to do perform multiple experiments in a relatively short time period.

Staining of lung sections from patients with cystic fibrosis or lung emphysema for ceramides showed that ceramide formation is increased in the airway epithelium [20]. By immunofluorescent staining we indeed found increased ceramides in the airway epithelium, in agreement with the increased ceramide levels in BAL which mainly contains products from epithelial cells. We also found positive immunofluorescence signals for ceramide in alveolar macrophages, which could be due to uptake of apoptotic alveolar epithelial cells, or internal ceramide production [42], triggered by the inflammatory response after elastase.

Sphingomyelinase inhibition has been examined in a variety of animal models. In a model of cystic fibrosis, acid sphingomyelinase inhibitors (desipramine and amitriptyline) and glycosphingolipid inhibitors (Miglustat) decreased pulmonary inflammation [14,43]. In spinal cord injury an inhibitor of acid sphingomyelinase (NB6) and an inhibitor of *de novo* synthesis of ceramide (Fumonisin B1) reduced spinal cell apoptosis and inflammation after injury, and improved motor function [44]. Zoledronic acid has been shown to specifically inhibit acid sphingomyelinase [45]. Sphingolactone has previously shown to ameliorate LPS-induced acute lung injury in mice by inhibiting neutral sphingomyelinase, thereby decreasing ceramide levels In the present study, treatment with myriocin (SPT inhibitor) produced a marked reduction in overall sphingolipid levels, which suggests that *de-novo* ceramide synthesis was responsible for their increase. The *de novo* pathway was also implicated in VEGF-inhibition-induced emphysema, demonstrating the importance of *de novo* ceramide generation in the process of alveolar destruction [33]. This is further corroborated by the observation that neither acid- nor neutral sphingomyelinase inhibitors were able to block the increase in ceramides. Inadequate dosing of these inhibitors seems unlikely since similar doses have been used successfully in other animal models [46-48]. Since inhibition of synthesis of very long acyl chain (C22-C24) ceramides in Cer synthase 2 knockout mice has been shown to elevate C16-ceramide and sphinganine levels [49], it is possible that inhibition of one specific step in the complex ceramide pathway leads to unintended alterations in individual ceramide metabolites. In the present study, desipramine exhibited an unexpected effect, namely upregulation of very long chain ceramides in both vehicle and elastase-treated mice. However, zoledronic acid, another inhibitor of acid sphingomyelinase, did not have this effect. Desipramine has previously been described to not only inhibit ASMase, but also acid ceramidase and other lysosomal enzymes [45,50]. This non-specific inhibitory effect could account for the different effect on sphingolipid levels compared to zoledronic acid. Acid sphingomyelinase protein levels were transiently upregulated after elastase instillation, suggesting that it may have contributed to the transient increase in ceramides. However, the contribution must have been minimal as we found no effect of acid sphingomyelinase inhibitors. The increased ceramide levels after elastase instillation could also be due to reduced degradation by ceramidases. A major role in the transient rise in ceramides, however, is unlikely since ceramide returned to normal 5 days after elastase instillation while acid ceramidase levels were still decreasing. The initial decrease of neutral ceramidase after elastase instillation may have contributed to the early increase in ceramide levels but neutral ceramidase levels were already increased and normalized at the time (day 2) ceramide levels peaked. Thus, contribution of neutral ceramidase to the elevation in ceramides after elastase treatment is most likely minimal.

Blocking the *de-novo* pathway of ceramide synthesis by myriocin had a positive effect on lung function but no effect on histology. A possible explanation for this structure-function discrepancy is that ceramides reduce surfactant synthesis, due to downregulation of Thyroid Transcription Factor-1 (TTF-1), and this may affect lung function without a measurable morphological alteration [24,51]. However, our observation of no changes in saturated phosphatidylcholine content in BAL of elastase-treated and control mice (data not shown) makes this explanation less likely. Clinical studies in humans also show that structural abnormalities of the lungs and airways are at best weakly correlated with changes in lung function, and this is especially true for localized or inhomogeneous structural abnormalities, as seen in our model [52,53]. Alternatively, the morphometric methodology may be insufficiently sensitive to demonstrate relatively small changes that might underlie significant functional improvement, or more sensitive approaches to the analysis of lung structure might be needed. Such approaches may include, but are not limited to, micro CT and advanced functional MRI imaging techniques.

It could be argued that desipramine, being a tricyclic antidepressant, and zoledronic acid, a biphosphonate, have limited specificity to influence ceramide metabolism. Desipramine is known to inhibit the re-uptake of norepinephrine and to a lesser extent serotonin [54]; however, both catecholamines are unlikely to affect ceramide levels in BAL. Zoledronic acid binds and blocks farnesyl diphosphate synthase in the HMG-CoA reductase pathway, preventing the formation of metabolites essential for sub-cellular protein trafficking [55]. As a bisphosphonate it may bind to calcium in bone, thereby preventing the functioning of osteoclasts, the only bone-resorbing cells in the body [56]. Again, it is very unlikely that these additional actions of zoledronic acid interfered with our ceramide outcomes. Importantly, we showed that neither desipramine nor zoledronic acid influenced BAL ceramide levels, which argues against a significant contribution of SMases in the production of ceramides in our elastase model [57].

In conclusion, we demonstrated the involvement of ceramides and dihydroceramides in elastase-induced emphysema. Our findings suggest an important role for ceramides in the acute phase of elastase-induced emphysema. Further experiments should be undertaken to better evaluate the preventive and therapeutic potencies of ceramide inhibition in this emphysema model, and ultimately in humans with COPD and emphysema.

## Abbreviations

BAL: Broncho-alveolar lavage; COPD: Chronic Obstructive Pulmonary Disease; SPT: Serine palmitoyltransferase; SMase: Sphingomyelinase; S1P: Sphingosine-1-phosphate; IF: Immunofluorescent; TTF-1: Thyroid Transcription Factor-1.

## Competing interests

Jeroen Tibboel declared no conflicts of interest relating to this manuscript. Professor Reiss declared no conflicts of interest relating to this manuscript. Professor de Jongste declared no conflicts of interest relating to this manuscript. Professor Post declared no conflicts of interest relating to this manuscript.

## Authors’ contributions

JT: acquisition and analysis of the data, writing of the manuscript and final approval of version to be published. IR: Involved in conception, writing of the manuscript and final approval of version to be published. JCdeJ: involved in conception and design of the study, writing of the manuscript and final approval of version to be published. MP: involved in conception and design of the study, writing of the manuscript and final approval of version to be published. All authors read and approved the final manuscript.

## Funding

This work was supported by an operating grant (MOP-86472) from the Canadian Institute of Health Research and infrastructure grants (CCURE, CSCCD) from the Canadian Foundation for Innovation.
